# Serum-Based Proteomics Reveals Lipid Metabolic and Immunoregulatory Dysregulation in Cervical Artery Dissection With Stroke

**DOI:** 10.3389/fneur.2020.00352

**Published:** 2020-05-19

**Authors:** Yongtao Yang, Jing Peng, Suxia Wang, Jialu Huang, Hong Ran, Kangning Chen, Zhenhua Zhou

**Affiliations:** ^1^Department of Neurology, Chongqing Renji Hospital, University of Chinese Academy of Sciences, Chongqing, China; ^2^Department of Geriatrics, Sichuan Second Hospital of Traditional Chinese Medicine, Chengdu, China; ^3^Department of Neurology, Southwest Hospital, Third Military Medical University, Chongqing, China

**Keywords:** cervical artery dissection, stroke, serum, immunoregulation, lipid metabolism

## Abstract

Cervical artery dissection (CAD) is an important causal factor for stroke in young and middle-aged individuals and presents a great burden to the individual stroke victim. However, the pathophysiological mechanisms underlying CAD remain unknown. Here, an iTRAQ (isobaric tagging for relative and absolute quantitation)–based quantitative proteomic approach was performed, to identify differentially expressed proteins in serum samples obtained from spontaneous CAD and non-CAD ischemic stroke subjects. Differential protein expression was analyzed for Kyoto Encyclopedia of Genes and Genomes (KEGG) pathway overrepresentation, and six differential proteins were selected for enzyme-linked immunosorbent assay validation. Through KEGG analysis, the significantly differentiated proteins were primarily involved in immunoregulation, blood coagulation, and lipid metabolism. For the first time, differential expressions of apolipoprotein B, apolipoprotein C-I, lipopolysaccharide-binding protein, vascular cell adhesion molecule 1, fibulin-1, and ficolin-2 were confirmed as being significantly upregulated in CAD as compared to non-CAD ischemic stroke subjects. In conclusion, proteomic analysis reveals that early perturbation of immunoregulation and lipid metabolism may be involved in the pathophysiology of CAD. Specifically, the panel of six proteins identified is promising as serum-based biomarkers for the detection of increased CAD risk in stroke subjects.

## Introduction

Cervical artery dissection (CAD) is an important cause of cerebral ischemia in young and middle-aged patients. Although accounting for only 2% of all ischemic strokes, CAD accounts for 8–25% of strokes in patients younger than 45 years (1). Such a high incidence of stroke presents a great burden not only to the individual stroke victim but also to their family, society, and the economy (2). A lack of clinical symptoms associated with CAD is regarded as the main cause of missed diagnoses. Thanks to the development of advanced imaging techniques, CAD has been increasingly recognized in the past few years as involved in at least some aspects of this disease. However, the underlying pathogenesis responsible for spontaneous CAD is unknown, making the diagnosis of CAD more challenging.

Unfortunately, the search for clinically useful predictors for stroke risk has been inconclusive. One study found that hyperhomocysteinemia and elevated peripheral leukocyte counts have been reported previously in patients with CAD. However, these factors have yielded poor results and lack specificity as a biomarker (3, 4). Currently, the candidate biomarkers for CAD risk that have shown promise include fibrillin-1 (5), serum neurofilament light chain (6), extracellular matrix (ECM)–regulating enzymes (7), and plasma TT MTHFR genotype (8). These factors, however, are neither standardized nor clinically practical as they exhibit low sensitivity and variable specificity.

To address this challenge, several lines of evidence suggest that the peripheral circulation may be adversely affected in individuals at risk of CAD and therefore may serve as a source of useful biomarkers for this disorder (9). Proteomics, the analysis of protein expression in biological samples, can improve our understanding of the pathophysiological mechanisms and aid in diagnostic tool development (10). We hypothesize that a proteomic signature for CAD can be detected in human serum.

In this study, serum samples from spontaneous CAD and non-CAD stroke subjects with ischemic events were analyzed using a quantitative proteomic approach based on isobaric tags for relative and absolute quantitation (iTRAQ) and multidimensional liquid chromatography–tandem mass spectrometry (LC-MS/MS). Differentially expressed proteins were further validated by enzyme-linked immunosorbent assay (ELISA) and analyzed by cluster Profiler R package bioinformatics resources. This proteomic approach may ultimately contribute to a better understanding of the pathophysiology underlying CAD.

## Materials and Methods

### Subjects and Ethics Statement

Fifteen patients with spontaneous CAD admitted to the Department of Neurology, Southwest Hospital, Army Medical University, from February 2014 to December 2016 were prospectively enrolled, all having symptoms of cerebral ischemia. Thirteen age- and sex-matched patients with ischemic stroke unrelated to CAD were admitted to our department during the same period for comparisons. The diagnosis of CAD was based on widely accepted criteria as previously described (11). Symptomatic patients with ischemic stroke within 15 days after the first symptom onset represented the acute stage. Patients with a recent history of head or neck trauma were excluded. Stroke severity was assessed based on the National Institutes of Health Stroke Scale (NIHSS) score. Dual antiplatelet treatment or anticoagulation therapy was utilized in all patients. The study was approved by the local ethics committee. Written informed consent was obtained from each patient or from an authorized family member.

### Sample Collection

All blood samples were obtained from non-fasting patients in the morning between 8:00 and 10:00 am. Serum samples were collected in 10-cc separator tubes (BD Vacutainers, catalog no. 367820; Becton, Dickinson & Co., Franklin Lakes, New Jersey, NJ, USA) and were kept at 4°C for 1 h and then centrifuged at 3,000 revolutions/min for 10 min at 4°C. The serum samples were distributed into 400-μL aliquots and stored at −80°C until use.

### Protein Digestion and iTRAQ Labeling

Each sample consisted of a pool of serum from 15 CAD and 13 non-CAD stroke subjects. The proteins in each sample were denatured, reduced, alkylated, and digested with sequencing-grade modified trypsin with a protein-to-enzyme ratio of 20:1 at 37°C overnight and then labeled with the following iTRAQ reagent tags in duplicate: 113 and 116 for CAD, and 115 and 118 for the non-CAD ischemic stroke subjects.

### LC-MS/MS Analysis for Peptide Identification and Sequence Database Search

The iTRAQ-labeled peptides were mixed and fractionated by 16 SCX chromatography using the AKTA Purifier system (GE Healthcare, Pittsburgh, PA, USA) and then for LC-MS/MS analysis using Q Exactive mass spectrometer (Thermo Fisher Scientific, Waltham, MA, USA). Desalted peptide mixture were loaded onto a Acclaim PePmap C18-reversed phase column (75 μm ×2 cm, 3 μm, 100 Å; Thermo Fisher Scientific, Waltham, MA, USA) and separated with reversed phase C18column (75 μm × 10 cm, 5 μm, 300 Å; Agela Technologies, Torrance, CA, USA) mounted onto a Dionex ultimate 3000 nano LC system (Thermo Fisher Scientific, Waltham, MA, USA). Peptides were eluted using a gradient of 5% to 80% (vol/vol) acetonitrile in 0.1% formic acid over 65 min at a flow rate of 400 nL min^−1^ combined with a Q Exactive mass spectrometer (Thermo Fisher Scientific, Waltham, MA, USA). The eluates were directly entered Q-Exactive MS (Thermo Fisher Scientific), setting in positive ion mode and data-dependent manner with full MS scan from 350 to 2,000 m/z, full scan resolution at 70,000, MS/MS scan resolution at 17,500. MS/MS scan with minimum signal threshold 1E+5, isolation width at 2 Da. To evaluate the performance of this mass spectrometry on the iTRAQ-labeled samples, two MS/MS acquisition modes, higher collision energy dissociation (HCD) was employed. And to optimize the MS/MS acquisition efficiency of HCD, normalized collision energy was systemically examined 28, stepped 20%.

All LC-MS/MS data were processed using Proteome Discoverer Software 1.3 (Thermo Fisher Scientific, Waltham, MA, USA). MS/MS spectra were searched using MASCOT engine (Matrix Science, London, UK; version 2.3) against the 2019-uniprot-human9606.fasta (downloaded 2011-11-11). For protein identification, the following options were used: peptide mass tolerance, 15 ppm; MS/MS tolerance, 20 mmu; enzyme, trypsin; max missed cleavage, 1; fixed modification, carbamidomethyl (C); variable modification, Oxidation(M) CGln → Pyro-Glu (N-term Q), iTRAQ8plex (K), iTRAQ 8 plex (Y), iTRAQ8plex (N-term); variable modification, oxidation (M); and decoy database pattern, reverse, and false discovery rate (FDR) <0.01.

### Gene Ontology and Pathway Analysis

To obtain ENTREZ Gene IDs, the org.Hs.eg.db R package of Bioconductor was used to search ENTREZ Gene IDs with UniProtKB accession and gene name. Then, using ENTREZ Gene IDs, gene ontology (GO) functional classification and enrichment analyses were performed to identify GO terms that were significantly enriched in differentially expressed proteins using the clusterProfiler R package (12). Furthermore, Kyoto Encyclopedia of Genes and Genomes (KEGG) pathway enrichment analysis was also performed to obtain enriched pathways using the clusterProfiler R package. To obtain further KEGG pathway information, a bioconductor package for pathway-based data integration and visualization was used (13).

### Enzyme-Linked Immunosorbent Assay Validation

A total of 28 individual serum samples consisting of CAD (*n* = 15) and non-CAD ischemic stroke (*n* = 13) subjects were employed in the ELISA validation. Six proteins including lipopolysaccharide-binding protein (LBP), vascular cell adhesion molecule 1 (VCAM1), ficolin-2 (FCN2), fibulin-1 (FBLN1), apolipoprotein C-I (APOC-I), and apolipoprotein B (APOB) were analyzed using commercially available kits (Wuhan Colorful Gene Biological Technology, Wuhan, China) following the manufacturers' instructions.

The reason why we selected the six proteins is that mounting evidence suggests a close association between these six proteins and key biological processes underlying CAD. In addition, we should consider the following factors: first, there is a statistically significant difference in expression level of these proteins between CAD and non-CAD group. Second, the result of MS/MS is very reliable. Third, the result from bioinformatics analysis indicated that six proteins might play key role in CAD. Every protein was analyzed in duplicate.

### Statistical Analysis

Statistical analysis was performed using the Statistical Package of Social Science (SPSS) for Windows v21.0 (SPSS, Chicago, IL, USA). Data are expressed as mean ± SD. The Student *t* test was applied to identify proteins with significant differences in abundance between the CAD and non-CAD ischemic stroke subjects. All the tests were two-tailed, and the significance threshold was set at *P* < 0.05.

We further evaluated the classification performance and the robustness of panel identification, as well as the six selected proteins using leave-one-out (LOO) cross-validation to alleviate overfitting of classifier training on small size data set. At each step of this procedure, one sample was left out for test, and the remaining 27 samples were used to build the LR classifiers. This process was repeated until all samples were selected as the test set once. So, these processes were cycled for 28 times. In the meantime, the predicted probabilities for each sample being CAD were obtained, from which we draw the receiver operating characteristic (ROC) curves (14). The optimal cutoff points were selected based on the Youden index and should be larger than 0.5.

## Results

### Subject Characteristics

Demographic and clinical data obtained from patients with CAD (*n* = 15) and patients with non-CAD ischemic stroke (*n* = 13) are provided in Table 1. The two groups of subjects were not distinguishable by key demographic characteristics, including age and sex. Patients with CAD had a lower prevalence of traditional vascular risk factors. Headache and Horner syndrome were seen more frequently in the CAD group compared to the non-CAD ischemic stroke group. However, differences were not statistically significant. The frequency of internal carotid artery dissection (nine patients representing, 60%) was higher than vertebral artery dissection (five patients representing 33.3%). In addition, bilateral internal carotid artery dissection was found in one patient. No significant difference was found in the meantime from symptom onset to blood removal between the group of patients with CAD (13.40 ± 3.21 days) and the group of patients with non-CAD ischemic stroke (9.93 ± 1.96 days).

**Table 1:** The basic demographic and clinical characteristics of subjects.

	**CAD (*n* = 15)**	**Non-CAD (*n* = 13)**	***P*-value**
Age, mean ± SD, years	45.27 ± 3.87	48.87 ± 2.68	0.45
Male sex, *n* (%)	11 (73.3)	9 (60.0)	0.46
Hypertension, *n* (%)	5 (33.3)	8 (53.3)	0.28
Diabetes mellitus, *n* (%)	1 (6.7)	4 (26.7)	0.15
Hyperlipidemia, *n* (%)	3 (20)	6 (40)	0.25
Smoking, *n* (%)	4 (26.7)	7 (46.7)	0.27
**Symptoms at onset**, ***n*** **(%)**			
Headache or neck pain	7 (46.7)	3 (20)	0.13
Horner sign	2 (13.3)	1 (6.7)	0.56
Stroke	15(100)	13 (100)	0.27
NIHSS score on admission	4.07 ± 1.14	6.53 ± 0.71	0.07
**Site of dissection**, ***n*** **(%)**			
Internal carotid artery	9 (60)	n/a	
Vertebral artery	5 (33.3)	n/a	
Multiple dissections	1 (6.7)	n/a	
Time to serum sampling, days	13.40 ± 3.21	9.93 ± 1.96	0.12

*n/a, not applicable; multiple dissection includes: ICA and VA, bilateral ICA and bilateral VA; ICA, internal carotid artery; VA, vertebral artery*.

### Qualitative Results

The immunodepleted protein fractions were then profiled using iTRAQ coupled to an LC-MS/MS–based approach. The identified proteins in the CAD subjects were used for non-CAD ischemic stroke comparisons. The FDR calculated by searching the data against a decoy database was 1%. Data from the two MS repetitions identified 438 unique proteins according to the parameters set as described above. Differentially regulated proteins (1.2-fold change with *P* < 0.05) were selected for further analysis. These cutoffs were selected based on a literature search of the reproducibility of iTRAQ TM quantification (15). One hundred thirty proteins were found to be differentially expressed in the CAD as compared to non-CAD ischemic stroke subjects (Supplementary Table 1).

### Annotation of Identified Proteins

**Figure 1:**
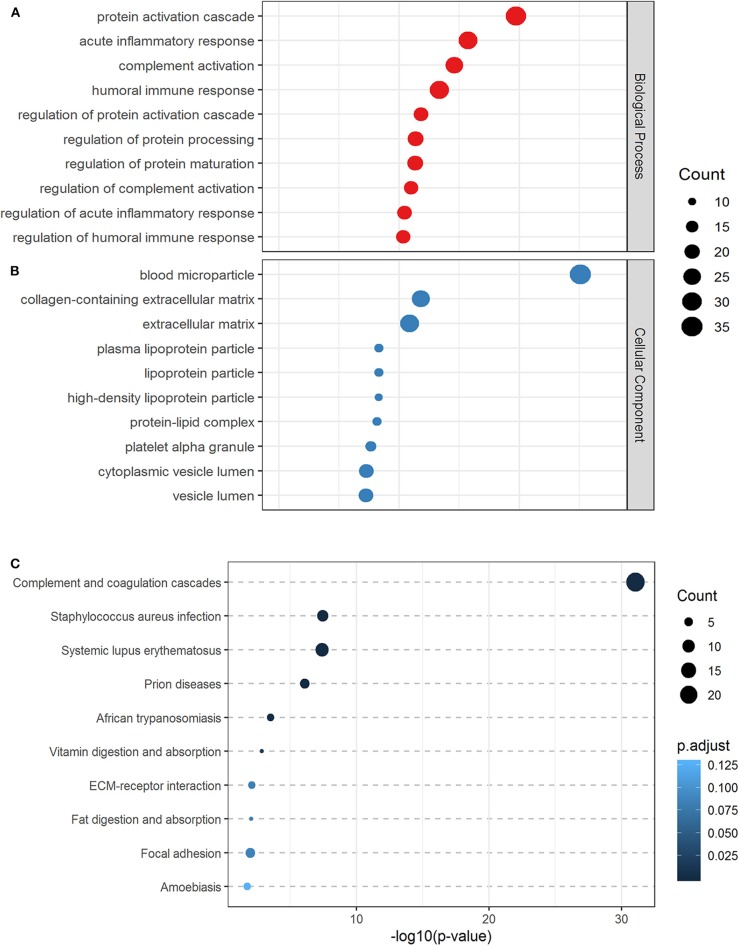
Gene ontology and pathway analysis. The top 10 biological processes **(A)** cellular components **(B)**, and canonical KEGG pathways **(C)** associated with differential proteins were based on gene ontology and pathway analysis.

The significantly differentiated proteins were categorized by biological function such as acute inflammatory response, humoral immune response, complement activation, and cellular component (blood microparticle, platelet alpha granule, plasma lipoprotein particle, lipoprotein particle, protein–lipid complex). The biologic processes (Figure 1A) and cellular component (Figure 1B) attributed to the differentially expressed proteins are displayed.

### Altered Biological Pathways Identified by KEGG

The 130 differentially expressed proteins were analyzed for KEGG overrepresentation of pathways. Inflammatory processes were the most statistically overrepresented, including complement and coagulation cascades ranking highest (*P* < 0.001) (Figure 1C).

### Detection of Differentially Expressed Proteins by ELISA

**Figure 2:**
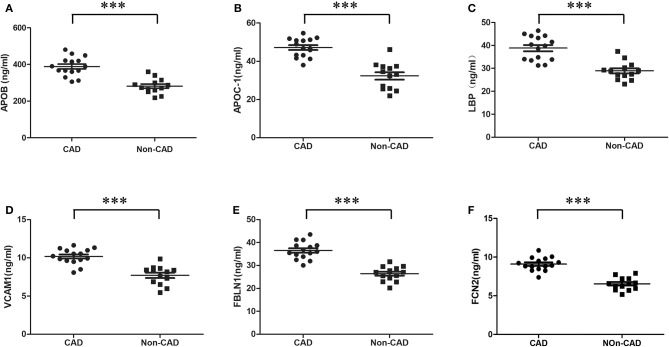
Enzyme-linked immunosorbent assay validation of differential proteins. Six proteins analyzed by ELISA displayed significant changes between CAD (*n* = 15) and non-CAD ischemic stroke (*n* = 13) subjects. **(A)** APOB, **(B)** APOC1, **(C)** LBP, **(D)** VCAM1, **(E)** FBLN1, and **(F)** FCN2 were significantly upregulated in CAD subjects compared to non-CAD ischemic stroke subjects. Statistical analysis was performed using the Statistical Package of Social Science (SPSS) for Windows v21.0. The Student *t* test was applied to identify proteins with significant differences in abundance between the CAD and non-CAD ischemic stroke subjects. All the tests were two-tailed, and the significance threshold was set at *P* < 0.05. ^***^P = 0.000. ELISA, enzyme-linked immunosorbent assay; CAD, cerebral artery dissection; APOB, apolipoprotein B; APOC-1, apolipoprotein C-I; LBP, lipopolysaccharide-binding protein; VCAM1, vascular cell adhesion molecule 1; FBLN1, fibulin-1; FCN2, ficolin-2.

Six candidate proteins (LBP, VCAM1, FCN2, FBLN1, APOC-I, and APOB) were assessed using individual samples from the CAD and non-CAD ischemic stroke subjects. Consistent with our proteomic findings, the expression levels of all proteins were significantly upregulated in CAD as compared to non-CAD ischemic stroke subjects (Figure 2).

### Diagnostic Performance

**Figure 3:**
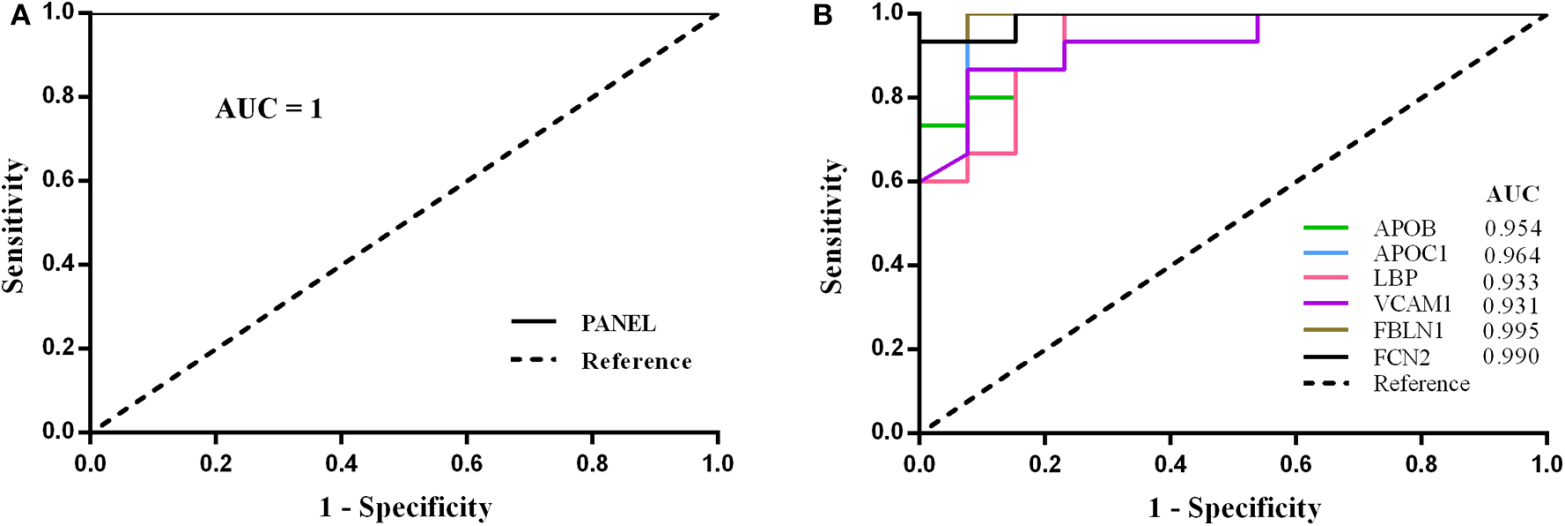
Receiver operating characteristic analyses showing the combined diagnostic utility of the panel including APOB, APOC1, LBP, VCAM1, FBLN1, and FCN2 in CAD. By leave-one-out (LOO) cross-validation, ELISA-based ROC analyses of combined quantitation of APOB, APOC1, LBP, VCAM1, FBLN1, and FCN2 showing promising for the detection of increased CAD risk in stroke subjects (PANEL = APOB + APOC1 + LBP + VCAM1 + FBLN1 + FCN2). The AUC of panel **(A)** was 1, and the panel exhibited a higher sensitivity (100%) and specificity (100%) than the six proteins individually **(B)**.

Based on ELISA results, all the six differentially expressed proteins were selected to form a biomarker panel. By LOO cross-validation, we got 28 formulas and 28 prediction scores listed in Supplementary Table 2. Receiver operating characteristic curves were constructed, in which the area under the curve (AUC) was 0.954, 0.964, 0.933, 0.931, 0.995, 0.990, and 1 for APOB, APOC-I, LBP, VCAM1, FBLN1, FCN2, and the whole panel, respectively (Figure 3). The whole panel exhibited a higher sensitivity (100%) and specificity (100%) than the six proteins individually: APOB (80.0%, 92.3%), APOC-I (93.3%, 92.3%), LBP (86.7%, 84.6%), VCAM1 (86.7%, 92.3%), FBLN1 (93.3%, 92.3%), and FCN2 (93.3%, 92.3%) (Table 2).

**Table 2:** Statistics of ROC curves.

**Variables**	**AUC**	**Cutoff**	***P*-value**	**Sensitivity**	**Specificity**	**95% confidence interval**	
						**Lower bound**	**Upper bound**
PANEL	1	0.500	0.000007	1	1	1	1
APOB	0.954	0.752	0.000046	0.800	0.923	0.886	1
APOC-I	0.964	0.4643	0.000031	0.933	0.923	0.898	1
LBP	0.933	0.469	0.000099	0.867	0.846	0.844	1
VCAM1	0.931	0.573	0.000109	0.867	0.923	0.838	1
FBLN1	0.995	0.483	0.000009	0.933	0.923	0.978	1
FCN2	0.990	0.701	0.000011	0.933	0.923	0.963	1

*ROC, receiver operating characteristic; AUC, area under the curve; APOB, apolipoprotein B; APOC-1, apolipoprotein C-I; LBP, lipopolysaccharide-binding protein; VCAM1, vascular cell adhesion molecule 1; FBLN1, fibulin-1; FCN2, ficolin-2; PANEL, APOB + APOC1 + LBP + VCAM1 + FBLN1 + FCN2*.

## Discussion

This study is the first reported proteomic-based study investigating putative peripheral biomarkers for CAD. Our analysis revealed immunoregulatory, coagulation, and lipid metabolic dysregulation in CAD, and the potential of using the panel of the six increased proteins with 100% sensitivity and 100% specificity shows to be promising for the detection of increased CAD risk in stroke subjects.

### Inflammatory Response

The complement and coagulation cascade were identified as a significantly altered pathway by KEGG analysis. Gene ontology annotation analysis demonstrated that the inflammatory response was identified as significantly altered (*P* < 0.01). Consistent with our findings, substantial evidence supported the view that dysfunction of the inflammatory response was well established in CAD. In the prospective multicenter CADISP (cervical artery dissection and ischemic stroke patients) trial, acute CAD was associated with particularly high white blood cell counts (4). An elevated leucocyte count was also described by Forster et al. (16) in a retrospective case control study comparing patients with CAD and traumatic CAD. Imaging studies have revealed signs of local inflammation around the affected vessels of patients with CAD, but not in those with traumatic CAD (17). However, the mechanism underlying the association between inflammation and occurrence of CAD remains speculative. It is proposed that inflammation in addition to constitutional connective tissue defects may increase the risk of arterial wall disintegration in CAD (4).

Lipopolysaccharide-binding protein, as a type I acute phase response protein, was considered as a marker for infections in stroke-associated pneumonia but with no data presented on microbiological findings (18). The present study demonstrated for the first time significantly increased levels of LBP in CAD compared to non-CAD ischemic stroke subjects, which were consistent with infection and inflammation as an important risk factor causing a transient arteriopathy linked to spontaneous CAD (19). Furthermore, we speculated that significantly increased LBP in CAD revealed the underlying imbalance of the immune system and increased the risk of infection in CAD subjects. However, the mechanism needs further study.

Vascular cell adhesion molecule 1, as a proinflammatory marker, plays a crucial role in the initiation of inflammatory mechanisms soon after cerebral damage and promotes migration of immune cells across the blood–brain barrier within the cerebral parenchyma (20). Vascular cell adhesion molecule 1 has been reported to be increased early after stroke (21) and may influence peripheral mechanisms such as cell adhesion–regulated processes and may lower nitric oxide (NO) release in the vessels, thereby contributing to decreased vascular endothelial function. Furthermore, VCAM1 has also been found to be associated with impaired endothelial dependent dilation in cerebral artery vasoreactivity, early after stroke with poor prognoses (22). In our study, the early increased level of VCAM1 may indicate an enhanced inflammatory state and more serious damage to endothelial function in CAD.

Human FCN2 is synthesized in the liver and secreted into the bloodstream where it is one of the few molecules known to activate the lectin pathway of complement (23) and may link complement to the coagulation system (24). Watanabe et al., have observed that serum levels of FCN2 in patients with systemic lupus erythematosus were significantly lower when compared to healthy controls, which is associated with thrombocytopenia (25). Although clinical research involving FCN2 is still in its infancy, evidence is emerging that suggests insufficiency of FCN2 may increase susceptibility to respiratory infections. In the present work, FCN2 was found to be upregulated in CAD subjects, demonstrating more susceptible to infection in the pathophysiology of CAD.

### Blood Coagulation

Gene ontology annotation analysis showed that coagulation-related components (blood microparticles and platelet alpha granule) were identified as significantly altered (*P* < 0.01). Inflammation may also impact on the coagulation system resulting in a hypercoagulable state, which may be responsible for ischemic events. Specifically, the physiological crosstalk between inflammation and coagulation may facilitate the formation of arterial and venous thrombosis, not only in the microcirculation, but also in larger vessels, a process that may be further aggravated by a preexisting or newly occurring vessel wall damage, as is the case in CAD (26). Pelz et al. (27) found evidence for a hypercoagulable state in patients with CAD as indicated by a shortened activated partial thromboplastin time, which was associated with a trend to an increased leucocyte count at the same time. Furthermore, the results strengthened the hypothesis that inflammation critically impacts on the occurrence of CAD and linked this condition to a marked effect on the coagulation system.

Fibulin-1, as an ECM protein, was found to be significantly upregulated in CAD as compared to stroke subjects in this study. In addition, FBLN1 has been found to bind to the plasma protein fibrinogen (FG) and to incorporate into fibrin clots formed *in vitro* and *in vivo* (28). It is possible that following vascular injury FBLN1 may be present in the extracellular matrix of the vessel wall and therefore may interact with plasma FG and promote platelet adhesion, leading to the formation of a platelet plug, and could therefore serve as a participant in the earliest events of thrombus formation (29). Mohamed et al. (30) showed that downregulation of FBLN1 may weaken extracellular components in the aorta and/or interfere with the transmission of cellular signals and eventually cause acute aortic dissection. Taken together, our findings supported the hypothesis that increased expression of FBLN1 may represent more serious blood vessel damage and a hypercoagulable state, which would be more likely to cause a more severe ischemic event in CAD subjects.

### Lipid Metabolism

Several studies have demonstrated that perturbation of lipid metabolism may be involved in the pathophysiology of both CAD (31) and stroke (32). Our bioinformatics analysis showed that lipid-related components (plasma lipoprotein particle, lipoprotein particle, protein–lipid complex) were identified as significantly altered (*P* < 0.01). This study was the first to report that APOB and APOC-I levels were significantly increased in CAD as compared to stroke subjects, indicating that the dysregulation of lipid metabolism may play an important role in the pathophysiology of CAD.

Apolipoprotein B present in very low-density lipoprotein (VLDL), intermediate-density lipoproteins, large buoyant low-density lipoprotein (LDL), and small dense LDL reflects the total number of atherogenic particles and leads to entrapment of these lipoproteins in the arterial wall. Recent reports suggest that the higher value of the APOB/APOA1 ratio, the more cholesterol is likely to be deposited in the arterial wall, thereby enhancing atherogenesis and increasing vascular risk (33). An additional study has supported the clinical utility of oxidized phospholipids-APOB as a biomarker for the prediction of recurrent fatal or nonfatal stroke, which have augmented proinflammatory responses and enhanced uptake of monocytes into the vessel wall (34). Here, the increased expression of APOB in CAD may indicate an enhanced vascular risk and more severe ischemic events.

Apolipoprotein C-I is a secreted plasma protein present in the circulation in association with LDL and VLDL and is produced by the liver. Kolmkova et al. (35) have shown that APOC-I and APOC-I–enriched high-density lipoprotein activated the neutral sphingomyelinase–ceramide signaling pathway, leading to apoptosis in human aortic smooth muscle cells, which may lead to plaque rupture *in vivo*. However, the potential physiological role and clinical significance of APOC-I in stroke have recently emerged. Allard et al. (36) first reported that APOC-I may be the plasma biomarker capable of accurately distinguishing between ischemic and hemorrhagic stroke in a small number of patients. Recent reports have shown that CAD-related infarcts are likely to be large when the vessel occludes and responds poorly to conventional treatment with antithrombotics, anticoagulants, or intravenous thrombolysis (37). In our study, we were the first to report increased APOC-I in CAD subjects, which may accelerate the plaque rupture, leading to a more severe stroke.

Finally, we conducted an LOO cross-validation to determine the predictive value of the panel combined with six proteins. This method enables ROC analysis to be performed even with the small sample sizes used in our study. We found the greatest predictive utility when combining measurements from our six upregulated proteins, resulting in high sensitivity (100%) and specificity (100%). However, the predicted performance had some limitations which were principally due to the small sample size. But the research gives us a hint that the panel of six proteins identified is promising for the detection of increased CAD risk in stroke subjects. We expect that, with further validation and research using a larger sample size, this panel will provide an early sensitive and specific predictor of CAD with stroke and will open a new avenue for the diagnosis of this disease.

### Limitations

The present study had some limitations. First, given the relatively small sample size of the study, some confounding effects could not be controlled, and this raised the risk of chance findings. Furthermore, the diagnostic efficiency of the model was reduced by the small sample size. Research with larger sample sizes and using healthy controls is necessary for a better comparison. Second, the timeline for this study was limited to acute dissection; subacute and chronic dissections were neglected. In future studies, a longer timeline, such as a 3 months' follow-up after acute stroke, should be implemented. Third, CAD subjects singly manifested with local signs (Horner syndrome and cranial nerve palsy), pain (cervical pain and headache), and transient ischemic attack (TIA) should be included for increased model diagnostic power. Furthermore, to elucidate the pathogenesis, subgroup analysis is necessary. In future studies, patients should be subdivided further on the basis of the following: clinical severity assessed with the (NIHSS) score; radiologic severity, based on the number of dissected arteries and luminal changes for each vessel; different therapeutic approaches with respect to strokes; delayed stroke upon CAD; dissection with and without ischemic events; and patients presented with ischemic events and hemorrhagic strokes. Further studies are needed to understand the mechanism(s) underlying CAD and to develop more diagnostically accurate biomarkers for stroke.

## Conclusion

In this study, 130 differentially expressed proteins were found in serum sampled from CAD and non-CAD ischemic stroke subjects using an iTRAQ-based proteomic approach. Most of these proteins were associated with inflammatory reactions, lipid metabolism, and coagulation cascades, suggesting that these processes were involved in the pathophysiology of CAD. Through ELISA validation, six differentially expressed proteins were found to be significantly upregulated in CAD relative to non-CAD ischemic stroke subjects. The panel containing the six proteins showed a higher sensitivity and specificity for the prediction of CAD. Further investigation of these proteins in a larger cohort of patients is now needed in order to better understand the underlying pathophysiology of CAD.

## Data Availability Statement

The raw data supporting the conclusions of this article will be made available by the authors, without undue reservation.

## Ethics Statement

The studies involving human participants were reviewed and approved by The Ethical Committee of Third Military Medical University. The patients/participants provided their written informed consent to participate in this study.

## Author Contributions

ZZ conceived and designed the research. YY did the statistical analysis and wrote the manuscript. JP made subject recruitment and collected the clinical data. JH collected the blood samples of patients and stored the serum samples. HR and SW performed the ELISA validations. KC made the LC-MS/MS analysis and annotated the functions of differentially expressed proteins. All authors reviewed the manuscript before submission.

## Conflict of Interest

The authors declare that the research was conducted in the absence of any commercial or financial relationships that could be construed as a potential conflict of interest.
