# Supplementary material for: Serum-Based Proteomics Reveals Lipid Metabolic and Immunoregulatory Dysregulation in Cervical Artery Dissection With Stroke
Source: Front Neurol. 2020 May 19;11:352. doi: 10.3389/fneur.2020.00352 (PMC7248409; doi:10.3389/fneur.2020.00352)
Supplement: Supplementary file 1 [file Table_1.DOCX]

**Supplementary table 1. Differential proteins identified by iTRAQ coupled with LC-MS/MS.**

| Accession No. | Gene name | Protein name | | Peptide count | | Unique peptide count | | Cover (%) | | Biological function | | Fold change  (CAD /non-CAD) | |
| --- | --- | --- | --- | --- | --- | --- | --- | --- | --- | --- | --- | --- | --- |
| F8VXY3 | OAS1 | 2'-5'-oligoadenylate synthase 1 | 1 | | 1 | | 3.06 | | immune response | | -8.69 | |  |
| Q6GMW4 | IGL@ | IGL@ protein | 10 | | 1 | | 49.79 | | immune response | | -2.81 | |  |
| A0A087WZB5 | PARVB | Beta-parvin | 1 | | 1 | | 4.15 | | [actin cytoskeleton reorganization](https://www.ebi.ac.uk/QuickGO/term/GO:0031532) | | -2.75 | |  |
| Q99543 | DNAJC2 | DnaJ homolog subfamily C member 2 | 1 | | 1 | | 1.29 | | [DNA replication](https://www.ebi.ac.uk/QuickGO/term/GO:0006260) | | -2.52 | |  |
| A1L196 | TUBA1B | Tubulin beta chain (Fragment) | 1 | | 1 | | 5.3 | | [microtubule-based process](https://www.ebi.ac.uk/QuickGO/term/GO:0007017) | | -2.47 | |  |
| B1AH77 | RAC2 | Ras-related C3 botulinum toxin substrate 2 | 3 | | 3 | | 15.54 | | [small GTPase mediated signal transduction](https://www.ebi.ac.uk/QuickGO/term/GO:0007264) | | -1.47 | |  |
| Q02985 | CFHR3 | Complement factor H-related protein 3 | 9 | | 3 | | 23.94 | | immune response | | -1.47 | |  |
| D3JV41 | N/A | C-X-C motif chemokine | 4 | | 4 | | 38.1 | | immune response | | -1.46 | |  |
| A2N011 | N/A | Vh1-D-J3-region | 2 | | 1 | | 14.96 | | immune response | | -1.44 | |  |
| A0A5E4 | N/A | Uncharacterized protein | 11 | | 1 | | 49.36 | | immune response | | -1.43 | |  |
| H3BVI7 | CDH1 | Cadherin-1 | 3 | | 3 | | 6.67 | | [homophilic cell adhesion](https://www.ebi.ac.uk/QuickGO/term/GO:0007156) | | -1.42 | |  |
| A2J1N2 | N/A | Rheumatoid factor RF-IP18 | 4 | | 1 | | 46.94 | | immune response | | -1.42 | |  |
| A0A0F7T8I6 | IGHV4-31 | IGHV4-31 protein | 1 | | 1 | | 9 | | immune response | | -1.41 | |  |
| S6BGE0 | N/A | IgG H chain | 7 | | 1 | | 23 | | immune response | | -1.40 | |  |
| Q0ZCH4 | N/A | Immunglobulin heavy chain variable region | 7 | | 4 | | 58.12 | | immune response | | -1.40 | |  |
| P13671 | C6 | Complement component C6 | 29 | | 3 | | 38.01 | | immune response | | -1.34 | |  |
| P67936 | TPM4 | Tropomyosin alpha-4 chain | 10 | | 8 | | 33.87 | | [muscle contraction](https://www.ebi.ac.uk/QuickGO/term/GO:0006936) | | -1.34 | |  |
| A0A120HG46 | N/A | GCT-A10 heavy chain variable region | 3 | | 1 | | 25.2 | | [muscle contraction](https://www.ebi.ac.uk/QuickGO/term/GO:0006936) | | -1.29 | |  |
| A0A125U0V2 | N/A | GCT-A1 heavy chain variable region | 3 | | 1 | | 25.2 | | immune response | | -1.27 | |  |
| B3KQK3 | N/A | Calumenin | 1 | | 1 | | 5.88 | | [cellular protein metabolic process](https://www.ebi.ac.uk/QuickGO/term/GO:0044267) | | -1.26 | |  |
| Q9BRL5 | N/A | CALM3 protein | 3 | | 3 | | 28.57 | | [calcium-mediated signaling](https://www.ebi.ac.uk/QuickGO/term/GO:0019722) | | -1.22 | |  |
| B2R8I | N/A | histidine-rich glycoprotein | 15 | | 15 | | 32.38 | | [angiogenesis](https://www.ebi.ac.uk/QuickGO/term/GO:0001525) | | -1.22 | |  |
| P02766 | TTR | Transthyretin | 10 | | 10 | | 73.47 | | [cellular protein metabolic process](https://www.ebi.ac.uk/QuickGO/term/GO:0044267) | | -1.22 | |  |
| P55083 | MFAP4 | Microfibril-associated glycoprotein 4 | 1 | | 1 | | 7.06 | | [cell adhesion](https://www.ebi.ac.uk/QuickGO/term/GO:0007155) | | -1.21 | |  |
| P00739 | HPR | Haptoglobin-related protein | 18 | | 8 | | 56.6 | | immune response | | -1.20 | |  |
| B0YJC4 | VIM | Vimentin | 10 | | 9 | | 23.67 | | [astrocyte development](https://www.ebi.ac.uk/QuickGO/term/GO:0014002) | | 1.20 | |  |
| P80108 | GPLD1 | Phosphatidylinositol-glycan-specific phospholipase D | 17 | | 17 | | 25.71 | | [cell migration involved in sprouting angiogenesis](https://www.ebi.ac.uk/QuickGO/term/GO:0002042) | | 1.20 | |  |
| P01031 | C5 | Complement C5 | 57 | | 57 | | 37.83 | | immune response | | 1.21 | |  |
| F5GX11 | PSMA1 | Proteasome subunit alpha type-1 | 1 | | 1 | | 5.04 | | [proteolysis involved in cellular protein catabolic process](https://www.ebi.ac.uk/QuickGO/term/GO:0051603) | | 1.21 | |  |
| A0A0S2Z3V0 | APOE | Apolipoprotein E isoform 2 | 17 | | 1 | | 75.13 | | lipid metabolic process | | 1.21 | |  |
| P05090 | APOD | Apolipoprotein D | 8 | | 8 | | 38.1 | | lipid metabolic process | | 1.21 | |  |
| Q15485 | FCN2 | Ficolin-2 | 7 | | 6 | | 23.64 | | immune response | | 1.22 | |  |
| H0YDL2 | WLS | Protein wntless homolog | 1 | | 1 | | 5..26 | | [Wnt signaling pathway](https://www.ebi.ac.uk/QuickGO/term/GO:0016055) | | 1.22 | |  |
| A0A087WYJ9 | IGHM | Ig mu chain C region | 29 | | 5 | | 62.17 | | immune response | | 1.23 | |  |
| P00747 | PLG | Plasminogen | 43 | | 43 | | 60.86 | | [blood coagulation](https://www.ebi.ac.uk/QuickGO/term/GO:0007596) | | 1.23 | |  |
| P51884 | LUM | Lumican | 12 | | 12 | | 42.9 | | [cartilage development](https://www.ebi.ac.uk/QuickGO/term/GO:0051216) | | 1.24 | |  |
| P02647 | APOA1 | Apolipoprotein A-I | 40 | | 40 | | 86.52 | | lipid metabolic process | | 1.24 | |  |
| P27169 | PON1 | Serum paraoxonase/arylesterase 1 | 15 | | 13 | | 60.0 | | [aromatic compound catabolic process](https://www.ebi.ac.uk/QuickGO/term/GO:0019439) | | 1.24 | |  |
| P00734 | F2 | Prothrombin | 41 | | 41 | | 62.38 | | blood coagulation | | 1.25 | |  |
| P55774 | CCL18 | C-C motif chemokine 18 | 1 | | 1 | | 24.72 | | immune response | | 1.26 | |  |
| B4DPP8 | N/A | highly similar to Kininogen-1 | 17 | | 17 | | 45.78 | | [blood coagulation](https://www.ebi.ac.uk/QuickGO/term/GO:0007597) | | 1.27 | |  |
| P05154 | SERPINA5 | Plasma serine protease inhibitor | 13 | | 13 | | 34.48 | | [blood coagulation](https://www.ebi.ac.uk/QuickGO/term/GO:0007596) | | 1.28 | |  |
| A0A024R462 | FN1 | Fibronectin 1 | 95 | | 95 | | 55.24 | | immune response | | 1.31 | |  |
| A0A024R1Q4 | APOL1 | Apolipoprotein L | 12 | | 12 | | 32.41 | | lipid metabolic process | | 1.31 | |  |
| P08697 | SERPINF2 | Alpha-2-antiplasmin | 19 | | 19 | | 46.84 | | immune response | | 1.32 | |  |
| P07357 | C8A | Complement component C8 alpha chain | 18 | | 18 | | 40.75 | | immune response | | 1.33 | |  |
| P04180 | LCAT | Phosphatidylcholine-sterol acyltransferase | 6 | | 6 | | 19.77 | | lipid metabolic process | | 1.33 | |  |
| A8K8Z4 | N/A | cDNA FLJ78071 | 27 | | 1 | | 35.01 | | immune response | | 1.33 | |  |
| P06727 | APOA4 | Apolipoprotein A-IV | 39 | | 39 | | 77.53 | | lipid metabolic process | | 1.35 | |  |
| A5YAK2 | APOC4 | Apolipoprotein C-IV | 5 | | 3 | | 29.92 | | lipid metabolic process | | 1.35 | |  |
| S6BGD4 | N/A | IgG H chain | 4 | | 1 | | 17.09 | | immune response | | 1.36 | |  |
| B2R950 | N/A | cDNA | 26 | | 18 | | 22.13 | | [DNA replication](https://www.ebi.ac.uk/QuickGO/term/GO:0006260) | | 1.39 | |  |
| B0YJ74 | PSMA8 | Proteasome subunit alpha type | 2 | | 2 | | 11.79 | | [interleukin-1-mediated signaling pathway](https://www.ebi.ac.uk/QuickGO/term/GO:0070498) | | 1.40 | |  |
| Q5T985 | ITIH2 | Inter-alpha-trypsin inhibitor heavy chain H2 | 32 | | 32 | | 41.28 | | [hyaluronan metabolic process](https://www.ebi.ac.uk/QuickGO/term/GO:0030212) | | 1.40 | |  |
| A0A087X1J7 | GPX3 | Glutathione peroxidase | 11 | | 11 | | 44.89 | | [response to oxidative stress](https://www.ebi.ac.uk/QuickGO/term/GO:0006979) | | 1.41 | |  |
| Q53TA7 | EFEMP1 | Putative uncharacterized protein EFEMP1 | 10 | | 10 | | 37.5 | | [epidermal growth factor receptor signaling pathway](https://www.ebi.ac.uk/QuickGO/term/GO:0007173) | | 1.42 | |  |
| B7Z550 | C8B | Complement component 8 | 20 | | 20 | | 48.58 | | immune response | | 1.42 | |  |
| Q03591 | CFHR1 | Complement factor H-related protein 1 | 14 | | 3 | | 43.94 | | immune response | | 1.43 | |  |
| A0A024R035 | C9 | Complement component 9 | 23 | | 23 | | 39.71 | | immune response | | 1.43 | |  |
| P04406 | GAPDH | Glyceraldehyde-3-phosphate dehydrogenase | 9 | | 9 | | 34.63 | | [gluconeogenesis](https://www.ebi.ac.uk/QuickGO/term/GO:0006094) | | 1.43 | |  |
| P02671 | FGA | Fibrinogen alpha chain | 25 | | 25 | | 38.68 | | blood coagulation | | 1.44 | |  |
| C0JYY2 | APOB | Apolipoprotein B | 226 | | 176 | | 53.63 | | lipid metabolic process | | 1.44 | |  |
| A2JA19 | N/A | Anti-mucin1 light chain variable region | 2 | | 2 | | 31.78 | | [DNA damage response](https://www.ebi.ac.uk/QuickGO/term/GO:0006977) | | 1.44 | |  |
| P01024 | C3 | Complement C3 | 138 | | 129 | | 83.64 | | immune response | | 1.45 | |  |
| A0A140VKE5 | QSCN6 | Sulfhydryl oxidase | 19 | | 19 | | 34.77 | | [cell redox homeostasis](https://www.ebi.ac.uk/QuickGO/term/GO:0045454) | | 1.46 | |  |
| K7ERI9 | APOC1 | Apolipoprotein C-I | 6 | | 6 | | 40.26 | | lipid metabolic process | | 1.48 | |  |
| Q1L857 | N/A | Ceruloplasmin | 43 | | 43 | | 51.69 | | [cellular iron ion homeostasis](https://www.ebi.ac.uk/QuickGO/term/GO:0006879) | | 1.48 | |  |
| P23142 | FBLN1 | Fibulin-1 | 25 | | 9 | | 45.66 | | [blood coagulation](https://www.ebi.ac.uk/QuickGO/term/GO:0072378) | | 1.48 | |  |
| V9H0D6 | C4A | Complement C4A3 | 1 | | 1 | | 28.95 | | immune response | | 1.48 | |  |
| L8E853 | VWF | von Willebrand factor | 19 | | 19 | | 9.39 | | [blood coagulation](https://www.ebi.ac.uk/QuickGO/term/GO:0007596) | | 1.48 | |  |
| B2RMS9 | ITIH4 | Inter-alpha (Globulin) inhibitor H4 | 34 | | 34 | | 43.12 | | [hyaluronan metabolic process](https://www.ebi.ac.uk/QuickGO/term/GO:0030212) | | 1.48 | |  |
| B4E1C4 | N/A | Vitamin K-dependent protein C | 11 | | 11 | | 32.78 | | [blood coagulation](https://www.ebi.ac.uk/QuickGO/term/GO:0007596) | | 1.51 | |  |
| P10909 | CLU | Clusterin | 23 | | 23 | | 42.98 | | immune response | | 1.52 | |  |
| P01008 | SERPINC1 | Antithrombin-III | 35 | | 35 | | 56.03 | | blood coagulation | | 1.53 | |  |
| P04004 | VTN | Vitronectin | 24 | | 24 | | 58.16 | | [cell adhesion](https://www.ebi.ac.uk/QuickGO/term/GO:0007155) | | 1.53 | |  |
| A0A024R9Q1 | THBS1 | Thrombospondin 1 | 45 | | 45 | | 44.7 | | immune response | | 1.54 | |  |
| Q65ZC9 | scFv | Single-chain Fv | 6 | | 2 | | 28.33 | | immune response | | 1.55 | |  |
| P35527 | KRT9 | Keratin | 29 | | 28 | | 53.29 | | [epidermis development](https://www.ebi.ac.uk/QuickGO/term/GO:0008544) | | 1.55 | |  |
| P00451 | F8 | Coagulation factor VIII | 6 | | 6 | | 3.28 | | blood coagulation | | 1.58 | |  |
| P02743 | APCS | Serum amyloid P-component | 12 | | 12 | | 39.46 | | immune response | | 1.57 | |  |
| Q6U2E7 | C4B | C4B1 | 3 | | 1 | | 80.33 | | immune response | | 1.58 | |  |
| A0A087X0P6 | IGKV2D-29 | Protein IGKV2D-29 | 3 | | 1 | | 43.14 | | immune response | | 1.58 | |  |
| Q5VY30 | RBP4 | Retinol binding protein 4 | 7 | | 7 | | 37.19 | | [cardiac muscle tissue development](https://www.ebi.ac.uk/QuickGO/term/GO:0048738) | | 1.63 | |  |
| B4DE89 | N/A | Tripeptidyl-peptidase 1 | 1 | | 1 | | 5.2 | | [central nervous system development](https://www.ebi.ac.uk/QuickGO/term/GO:0007417) | | 1.66 | |  |
| P05160 | F13B | Coagulation factor XIII B chain | 3 | | 3 | | 7.26 | | blood coagulation | | 7.26 | |  |
| P07225 | PROS1 | Vitamin K-dependent protein S | 25 | | 25 | | 40.83 | | blood coagulation | | 1.68 | |  |
| Q6ZW64 | N/A | cDNA FLJ41552 fis | 15 | | 2 | | 43.32 | | immune response | | 1.73 | |  |
| Q53HT9 | N/A | Complement component 1 | 26 | | 1 | | 49.65 | | immune response | | 1.75 | |  |
| P09871 | C1S | Complement C1s subcomponent | 25 | | 24 | | 43.02 | | immune response | | 1.78 | |  |
| H0YLI6 | IDH3A | Isocitrate dehydrogenase | 1 | | 1 | | 4.48 | | [glutathione metabolic process](https://www.ebi.ac.uk/QuickGO/term/GO:0006749) | | 1.79 | |  |
| H6VRF8 | KRT1 | Keratin 1 | 35 | | 32 | | 46.74 | | [epidermis development](https://www.ebi.ac.uk/QuickGO/term/GO:0008544) | | 1.82 | |  |
| P02760 | AMBP | Protein AMBP | 14 | | 14 | | 45.45 | | [cell adhesion](https://www.ebi.ac.uk/QuickGO/term/GO:0007155) | | 1.82 | |  |
| Q53GY3 | N/A | Angiotensinogen variant | 12 | | 8 | | 33.2 | | [activation of phospholipase C activity](https://www.ebi.ac.uk/QuickGO/term/GO:0007202) | | 1.83 | |  |
| A0A0B4J1X5 | IGHV3-74 | Protein IGHV3-74 | 5 | | 1 | | 41.88 | | immune response | | 1.85 | |  |
| A4D2D2 | PCOLCE | Procollagen C-endopeptidase enhance | 15 | | 15 | | 44.32 | | [cellular response to leukemia inhibitory factor](https://www.ebi.ac.uk/QuickGO/term/GO:1990830) | | 1.91 | |  |
| Q07507 | DPT | Dermatopontin | 2 | | 2 | | 15.92 | | [cell adhesion](https://www.ebi.ac.uk/QuickGO/term/GO:0007155) | | 1.91 | |  |
| F2RM37 | F9 | Coagulation factor IX | 10 | | 10 | | 28.2 | | blood coagulation | | 1.92 | |  |
| F8UU18 | VCAM1 | Vascular cell adhesion molecule 1 | 1 | | 1 | | 7.69 | | [cardiac neuron differentiation](https://www.ebi.ac.uk/QuickGO/term/GO:0060945) | | 1.93 | |  |
| P18428 | LBP | Lipopolysaccharide-binding protein | 16 | | 16 | | 29.94 | | immune response | | 1.93 | |  |
| Q96SB0 | N/A | Anti-streptococcal/anti-myosin immunoglobulin lambda light chain variable region | 2 | | 1 | | 22.22 | | immune response | | 1.94 | |  |
| P02741 | CRP | C-reactive protein | 9 | | 9 | | 29.02 | | immune response | | 1.97 | |  |
| P13645 | KRT10 | Keratin | 34 | | 30 | | 57.53 | | [epidermis development](https://www.ebi.ac.uk/QuickGO/term/GO:0008544) | | 2.00 | |  |
| A0A075B6H6 | IGKC | Ig kappa chain C region | 7 | | 5 | | 80.37 | | immune response | | 2.00 | |  |
| B0AZL7 | N/A | cDNA | 5 | | 5 | | 12.73 | | [DNA replication](https://www.ebi.ac.uk/QuickGO/term/GO:0006260) | | 2.04 | |  |
| C9JN98 | SERPINE2 | Glia-derived nexin (Fragment) | 1 | | 1 | | 3.64 | | [blood coagulation](https://www.ebi.ac.uk/QuickGO/term/GO:0007596) | | 2.09 | |  |
| A0A087WSY5 | CPB2 | Carboxypeptidase B2 | 12 | | 12 | | 34.977 | | [blood coagulation](https://www.ebi.ac.uk/QuickGO/term/GO:0007596) | | 2.09 | |  |
| A2J1M3 | N/A | Rheumatoid factor RF-ET5 | 2 | | 1 | | 21.88 | | immune response | | 2.11 | |  |
| P35908 | KRT2 | Keratin | 34 | | 28 | | 69.17 | | [epidermis development](https://www.ebi.ac.uk/QuickGO/term/GO:0008544) | | 2.11 | |  |
| V9HVX8 | HEL-109 | Epididymis luminal protein 109 | 9 | | 9 | | 8.72 | | [cerebral cortex development](https://www.ebi.ac.uk/QuickGO/term/GO:0021987) | | 2.17 | |  |
| D6RD58 | LECT2 | Leukocyte cell-derived chemotaxin-2 | 1 | | 1 | | 11.39 | | immune response | | 2.27 | |  |
| P62805 | HIST1H4A | Histone H4 | 7 | | 7 | | 58.52 | | [DNA replication](https://www.ebi.ac.uk/QuickGO/term/GO:0006335) | | 2.30 | |  |
| M0QZL1 | BLVRB | Flavin reductase (NADPH) | 1 | | 1 | | 9.74 | | Glucose metabolism | | 2.30 | |  |
| B4DPC6 | N/A | cDNA FLJ51022 | 2 | | 2 | | 4.63 | | [extracellular matrix organization](https://www.ebi.ac.uk/QuickGO/term/GO:0030198) | | 2.38 | |  |
| B7Z752 | N/A | cDNA FLJ50715 | 1 | | 1 | | 2.19 | | [protein import into nucleus](https://www.ebi.ac.uk/QuickGO/term/GO:0006606) | | 2.42 | |  |
| P01591 | JCHAIN | Immunoglobulin J chain | 6 | | 6 | | 54.72 | | immune response | | 2.51 | |  |
| Q5NV92 | V5-6 | V5-6 protein | 1 | | 1 | | 19.19 | | immune response | | 2.52 | |  |
| P14543 | NID1 | Nidogen-1 | 4 | | 4 | | 3.85 | | [basement membrane organization](https://www.ebi.ac.uk/QuickGO/term/GO:0071711) | | 2.53 | |  |
| A2NB45 | N/A | Cold agglutinin FS-1 L-chain | 3 | | 1 | | 38.94 | | immune response | | 2.58 | |  |
| P02747 | C1QC | Complement C1q subcomponent subunit C | 5 | | 5 | | 22.04 | | immune response | | 2.61 | |  |
| Q5UGI6 | SERPING1 | Serine/cysteine proteinase inhibitor clade G member 1 splice variant 2 | 4 | | 4 | | 14.11 | | immune response | | 2.61 | |  |
| A7L3A3 | AGT | Angiotensinogen | 5 | | 1 | | 71.59 | | [positive regulation of extrinsic apoptotic signaling pathway](https://www.ebi.ac.uk/QuickGO/term/GO:2001238) | | 2.85 | |  |
| P0C0L4 | C4A | Complement C4-A | 102 | | 3 | | 64.28 | | immune response | | 2.87 | |  |
| A0A087WXP0 | AZU1 | Azurocidin | 2 | | 1 | | 23.33 | | [defense response to virus](https://www.ebi.ac.uk/QuickGO/term/GO:0051607) | | 3.00 | |  |
| Q5NV79 | V5-4 | V5-4 protein | 1 | | 1 | | 19.19 | | immune response | | 3.14 | |  |
| D6RHH7 | CRHBP | Corticotropin-releasing factor-binding protein | 1 | | 1 | | 3.36 | | [inflammatory response](https://www.ebi.ac.uk/QuickGO/term/GO:0006954) | | 3.91 | |  |
| B4E3R1 | N/A | cDNA FLJ55924 | 1 | | 1 | | 1.65 | | [cytoskeletal anchoring at nuclear membrane](https://www.ebi.ac.uk/QuickGO/term/GO:0090286) | | 4.51 | |  |
| A0A0J9YWK7 | TRAPPC9 | Trafficking protein particle complex subunit 9 | 1 | | 1 | | 10.99 | | [cerebral cortex development](https://www.ebi.ac.uk/QuickGO/term/GO:0021987) | | 6.25 | |  |
| Q9UNU2 | C4B | Complement protein C4B frameshift mutant | 14 | | 1 | | 55.08 | | immune response | | 6.56 | |  |
| A0A087WSY6 | IGKV3D-15 | Protein IGKV3D-15 | 2 | | 1 | | 26.09 | | immune response | | 10.72 | |  |
| Q2VPK5 | CTU2 | Cytoplasmic tRNA 2-thiolation protein 2 | 1 | | 1 | | 1.75 | | [tRNA modification](https://www.ebi.ac.uk/QuickGO/term/GO:0006400) | | 29.60 | |  |
