# Supplementary material for: Serum-Based Proteomics Reveals Lipid Metabolic and Immunoregulatory Dysregulation in Cervical Artery Dissection With Stroke
Source: Front Neurol. 2020 May 19;11:352. doi: 10.3389/fneur.2020.00352 (PMC7248409; doi:10.3389/fneur.2020.00352)
Supplement: Supplementary file 2 [file Table_2.DOCX]

**Supplementary table 2. Statistics and Scores for integrating 6-proteins.**

| Group | APOB | APOB_coef | APOC-I | APOC-I_coef | LBP | LBP_coef | VCAM1 | VCAM1_coef | FBLN1 | FBLN1_coef | FCN2 | FCN2_coef | Constant | Scores |
| --- | --- | --- | --- | --- | --- | --- | --- | --- | --- | --- | --- | --- | --- | --- |
| Non-CAD | 341.351 | 0.056815 | 27.063 | 1.183796 | 24.795 | 2.299645 | 8.623 | -1.558151 | 24.474 | 0.641638 | 5.958 | 5.573897 | -192.675343 | -48.747926 |
| Non-CAD | 270.692 | 0.068473 | 34.521 | 1.042308 | 29.321 | 2.250777 | 7.593 | -2.04628 | 29.59 | 0.596748 | 5.184 | 6.715242 | -192.500137 | -35.052732 |
| Non-CAD | 225.668 | 0.068153 | 36.233 | 1.086681 | 25.094 | 2.284257 | 6.876 | -1.765505 | 27.061 | 0.579745 | 6.251 | 6.13411 | -192.661315 | -38.693317 |
| Non-CAD | 264.558 | 0.059032 | 24.426 | 1.18901 | 34.7 | 2.303838 | 8.706 | -1.479945 | 22.932 | 0.624388 | 7.229 | 5.364509 | -192.229203 | -27.411778 |
| Non-CAD | 287.87 | 0.063816 | 33.155 | 1.114074 | 31.543 | 2.278097 | 8.168 | -1.752358 | 27.091 | 0.612581 | 6.65 | 5.994992 | -192.152107 | -22.837389 |
| Non-CAD | 315.822 | -0.018424 | 37.223 | 0.938667 | 27.551 | 1.084993 | 9.838 | 0.199992 | 31.659 | 0.676346 | 7.917 | 7.877515 | -151.020623 | -6.2604381 |
| Non-CAD | 259.658 | 0.06201 | 25.819 | 1.248227 | 37.47 | 2.559079 | 8.38 | -0.583788 | 28.641 | 0.648843 | 6.578 | 2.002936 | -185.399125 | -14.314387 |
| Non-CAD | 290.327 | 0.063201 | 37.413 | 1.082296 | 28.96 | 2.261652 | 6.514 | -1.848926 | 20.238 | 0.648739 | 5.71 | 6.256512 | -192.57029 | -31.421999 |
| Non-CAD | 251.994 | 0.058542 | 25.517 | 1.229378 | 27.371 | 2.332678 | 8.426 | -1.267885 | 25.663 | 0.594433 | 7.214 | 4.985869 | -192.58226 | -42.072465 |
| Non-CAD | 274.065 | 0.031797 | 21.982 | 1.581548 | 30.341 | 2.471759 | 5.972 | 1.041651 | 29.686 | 0.53148 | 7.759 | 1.766267 | -196.271035 | -42.092641 |
| Non-CAD | 218.633 | 0.072779 | 46.174 | 0.994235 | 23.239 | 2.249392 | 8.274 | -2.252772 | 25.515 | 0.623698 | 6.243 | 6.932185 | -192.664906 | -38.019737 |
| Non-CAD | 360.445 | 0.05901 | 32.472 | 1.153787 | 26.891 | 2.288682 | 7.321 | -1.545056 | 27.717 | 0.616176 | 5.773 | 5.751687 | -192.619465 | -33.367202 |
| Non-CAD | 300.459 | 0.066607 | 38.123 | 1.128987 | 28.811 | 2.287557 | 5.453 | -1.882862 | 22.872 | 0.577087 | 6.578 | 5.934537 | -190.959472 | -20.030351 |
| CAD | 366.608 | 0.066082 | 43.144 | 1.100898 | 44.143 | 2.284676 | 9.488 | -1.893135 | 36.008 | 0.605948 | 9.497 | 6.148447 | -192.225635 | 42.598862 |
| CAD | 419.693 | 0.068587 | 46.75 | 1.040186 | 46.473 | 2.263412 | 8.479 | -2.314227 | 43.523 | 0.669486 | 10.055 | 6.723967 | -192.678963 | 67.0479588 |
| CAD | 458.986 | 0.064053 | 50.716 | 1.16859 | 34.82 | 2.310315 | 10.531 | -1.538743 | 38.717 | 0.582633 | 8.767 | 5.46327 | -192.121011 | 31.239585 |
| CAD | 390.046 | 0.054636 | 51.835 | 1.294881 | 41.514 | 2.357515 | 9.739 | -1.100044 | 41.223 | 0.605307 | 8.27 | 4.47622 | -192.427763 | 45.130406 |
| CAD | 305.683 | 0.047931 | 52.299 | 1.405864 | 39.853 | 2.394868 | 8.07 | -0.740233 | 33.816 | 0.58 | 7.379 | 3.7216965 | -192.250054 | 32.471592 |
| CAD | 360.444 | 0.070223 | 48.943 | 0.965465 | 33.496 | 2.256079 | 9.617 | -1.292272 | 35.347 | 0.573251 | 8.81 | 6.179487 | -192.790119 | 17.619919 |
| CAD | 480.677 | 0.073192 | 41.191 | 1.042546 | 44.264 | 2.278309 | 10.966 | -2.01569 | 38.627 | 0.575712 | 9.794 | 6.487221 | -192.67619 | 49.965916 |
| CAD | 389.121 | 0.049799 | 51.256 | 1.447142 | 43.266 | 2.43912 | 11.63 | -0.058301 | 31.898 | 0.456781 | 8.241 | 3.141325 | -193.794197 | 45.069335 |
| CAD | 312.134 | 0.100665 | 37.984 | 1.802756 | 40.396 | 1.90087 | 10.203 | 1.211811 | 34.746 | -0.294836 | 8.832 | 0.938301 | -179.455876 | 7.6353319 |
| CAD | 421.24 | 0.067904 | 45.867 | 1.069102 | 34.008 | 2.263093 | 10.5 | -1.984141 | 36.85 | 0.604078 | 9.925 | 6.412137 | -192.088079 | 27.582824 |
| CAD | 367.533 | 0.004983 | 41.574 | 1.230782 | 38.676 | 2.094585 | 11.248 | -2.984559 | 30.014 | 1.711088 | 9.302 | 4.795908 | -186.018064 | 10.389865 |
| CAD | 415.367 | 0.067729 | 50.832 | 1.044972 | 31.272 | 2.242964 | 9.838 | -2.169187 | 37.964 | 0.623588 | 10.863 | 6.764738 | -192.145557 | 35.065603 |
| CAD | 329.66 | 0.054996 | 54.658 | 1.280943 | 33.887 | 2.342785 | 10.927 | -1.031057 | 32.437 | 0.557553 | 9.222 | 4.68619 | -192.333074 | 25.235676 |
| CAD | 366.916 | 0.061877 | 43.221 | 1.196138 | 45.081 | 2.328604 | 9.937 | -1.41545 | 35.497 | 0.577292 | 8.464 | 5.243214 | -192.307732 | 37.875378 |
| CAD | 449.387 | -0.036352 | 46.75 | 1.55054 | 31.422 | 2.296668 | 11.318 | -0.929723 | 41.102 | 0.06 | 8.919 | 7.214266 | -177.7237 | 6.8813842 |

# Abbreviations: CAD, Cerebral artery dissection; APOB, Apolipoprotein B; APOC-1, Apolipoprotein C-I; LBP, Lipopolysaccharide-binding protein; VCAM1, Vascular cell adhesion molecule 1; FBLN1, Fibulin-1; FNC2, Ficolin-2; Coef , coefficient; Scores: APOB*APOB_coef + APOC*APOC_coef + LBP*LBP_coef + VCAM1* VCAM1_coef + FBLN1*FBLN1_coef + FCN2*FCN2_coef + constant.
